# Different Contacted Cell Types Contribute to Acquiring Different Properties in Brain Microglial Cells upon Intercellular Interaction

**DOI:** 10.3390/ijms24021774

**Published:** 2023-01-16

**Authors:** Akiko Nakano-Doi, Shuji Kubo, Emiko Sonoda, Akihiko Taguchi, Takayuki Nakagomi

**Affiliations:** 1 Institute for Advanced Medical Sciences, Hyogo Medical University, 1-1 Mukogawacho, Nishinomiya 663-8501, Japan; 2 Department of Therapeutic Progress in Brain Diseases, Hyogo Medical University, 1-1 Mukogawacho, Nishinomiya 663-8501, Japan; 3 Department of Regenerative Medicine Research, Foundation for Biomedical Research and Innovation at Kobe, 2-2 Minatojima Minamimachi, Chuo-ku, Kobe 650-0047, Japan

**Keywords:** astrocyte, cell–cell contact, endothelial cell, gap junction, microglial cell, pericyte

## Abstract

Microglial cells (MGs), originally derived from progenitor cells in a yolk sac during early development, are glial cells located in a physiological and pathological brain. Since the brain contains various cell types, MGs could frequently interact with different cells, such as astrocytes (ACs), pericytes (PCs), and endothelial cells (ECs). However, how microglial traits are regulated via cell–cell interactions by ACs, PCs, or ECs and how they are different depending on the contacted cell types is unclear. This study aimed to clarify these questions by coculturing MGs with ACs, PCs, or ECs using mouse brain-derived cells, and microglial phenotypic changes were investigated under culture conditions that enabled direct cell–cell contact. Our results showed that ACs or PCs dose-dependently increased the number of MG, while ECs decreased it. Microarray and gene ontology analysis showed that cell fate-related genes (e.g., cell cycle, proliferation, growth, death, and apoptosis) of MGs were altered after a cell–cell contact with ACs, PCs, and ECs. Notably, microarray analysis showed that several genes, such as gap junction protein alpha 1 (Gja1), were prominently upregulated in MGs after coincubation with ACs, PCs, or ECs, regardless of cell types. Similarly, immunohistochemistry showed that an increased Gja1 expression was observed in MGs after coincubation with ACs, PCs, or ECs. Immunofluorescent and fluorescence-activated cell sorting analysis also showed that calcein-AM was transferred into MGs after coincubation with ACs, PCs, or ECs, confirming that intercellular interactions occurred between these cells. However, while Gja1 inhibition reduced the number of MGs after coincubation with ACs and PCs, this was increased after coincubation with ECs; this indicates that ACs and PCs positively regulate microglial numbers via Gja1, while ECs decrease it. Results show that ACs, PCs, or ECs exert both common and specific cell type-dependent effects on MGs through intercellular interactions. These findings also suggest that brain microglial phenotypes are different depending on their surrounding cell types, such as ACs, PCs, or ECs.

## 1. Introduction

Microglial cells (MGs) are resident immune cells that exert multiple functions in a healthy and pathological brain. MGs are also involved in the pathogenesis of various diseases in the central nervous system, including ischemic stroke, traumatic brain injuries, Alzheimer’s disease, and brain tumors [1,2,3,4,5]. Therefore, insights into microglial regulation are essential in understanding the progress of such diseases.

In a healthy brain, MGs are widely distributed in part as a portion of the neurovascular unit (NVU) consisting of astrocytes (ACs), pericytes (PCs), and endothelial cells (ECs) [6,7,8]. PCs and ECs play important roles in maintaining the function of NVU by regulating the blood–brain barrier (BBB) permeability and cerebral blood flow (CBF) [9,10,11,12]. ACs can also regulate NVU by controlling ion and water concentrations via aquaporin 4 [13]. Although the exact role of MGs in NVUs of a healthy brain remains unclear, a recent study showed that MGs can partially regulate CBF by modulating vascular cyclic GMP levels [14], suggesting that brain MGs play an important role in NVU regulation under physiological conditions.

MGs could regulate damaged NVUs by modulating CBF and BBB integrity even in pathological cases [14,15]. However, under severe pathological conditions, such as after an ischemic stroke, NVUs could be disrupted, and certain MGs would accumulate within and around the ischemic areas [16,17]. ACs would also rapidly disappear from the ischemic areas after a stroke [17,18,19], indicating their sensitivity to hypoxia. However, ACs would emerge around the ischemic areas after a stroke [17,19]. Therefore, MGs and ACs could establish again direct cell–cell contacts around the ischemic areas. Unlike ACs, PCs and ECs are resistant to hypoxia and could survive within the ischemic areas for several days even after a permanent ischemia [17,20,21,22,23]. Furthermore, the number of PDGFRβ^+^PCs reportedly increases within the ischemic areas after an ischemic stroke [17,19]. These findings indicate that although NVUs might not be complete in certain pathological brains, ACs, PCs, and ECs are likely to keep surrounding MGs even after severe brain injuries.

Therefore, regardless of NVU conditions, ACs, PCs, and ECs could potentially localize near MGs in a physiological and pathological brain; they could also regulate microglial traits via cell–cell interactions. Although several studies described the crosstalk between MGs and other cell types, most of them focused on how MGs affected AC, PC, and EC traits [24,25,26]. Therefore, how these cells affect MG traits via cell–cell interactions and how microglial traits are different depending on the contacted cell types remain unclear.

In this study, using mouse brain-derived cells, MGs were coincubated with ACs, PCs, or ECs to investigate such effects. The phenotypic alternations of MGs after a direct cell–cell contact culture with the aforementioned cell types were also investigated using immunohistochemical, flow cytometric, and DNA microarray analyses.

## 2. Results

### 2.1. MG, AC, PC, and EC Expression Patterns in Healthy Brain Samples

**Figure 1:**
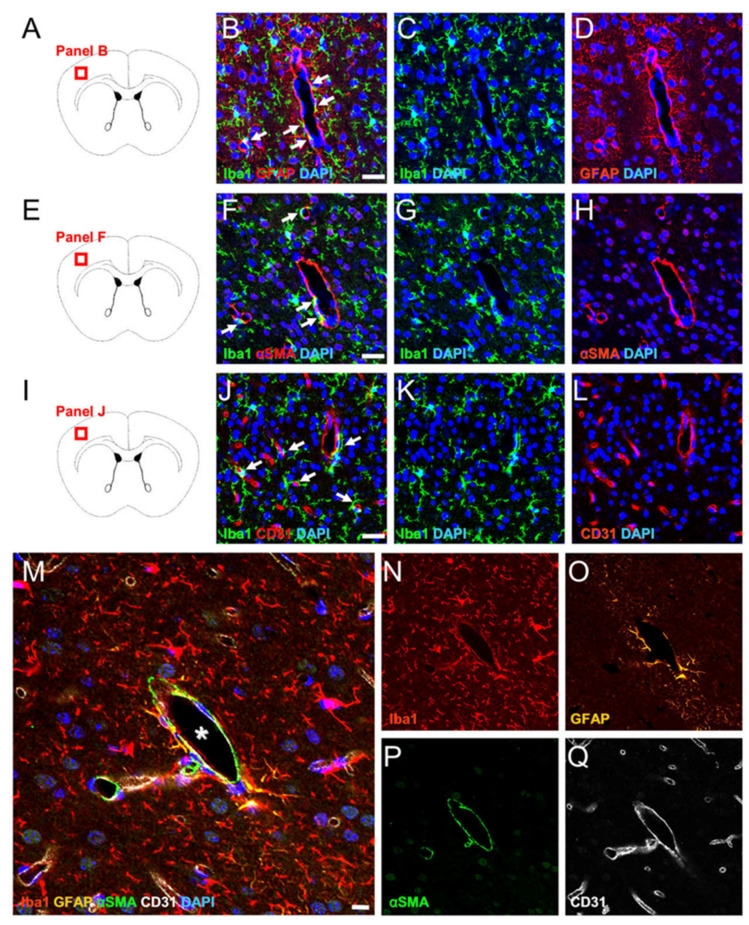
Localization of MGs, ACs, PCs, and ECs in healthy brain samples. Double immunohistochemical staining (**A**–**L**) for Iba1/GFAP [Iba1 ((**B**,**C**): green); GFAP ((**B**,**D**): green); DAPI ((**B**–**D**): blue)], Iba1/αSMA [Iba1 ((**F**,**G**): green); αSMA ((**F**,**H**): green); DAPI ((**F**–**H**): blue)], and Iba1/CD31 [Iba1 ((**J**,**K**): green); CD31 ((**J**,**L**): green); DAPI ((**J**–**L**): blue)] showed that Iba+ MGs were located near GFAP+ ACs (arrows in (**B**)), αSMA+ PCs (arrows in (**F**)), and CD31+ ECs (arrows in (**J**)) in normal mouse brain samples. Quadruple immunohistochemical staining (**M**–**Q**) also showed that Iba+ MGs ((**M**,**N**): red) around blood vessels (asterisk in (**M**)) were located near GFAP+ ACs ((**M**,**O**): orange), αSMA+ PCs ((**M**,**P**): green), and CD31+ ECs ((**M**,**Q**): white) in normal mouse brain samples. The results represent triplicates. Scale bars: 25 µm (**B**,**F**,**J**) and 10 µm (**M**). Abbreviations: AC, astrocyte; αSMA, alpha-smooth muscle actin; DAPI, 4’,6-diamidino-2-phenylindole; EC, endothelial cell; GFAP, glial fibrillary acidic protein; MG, microglial cell; PC, pericyte.

The brain is composed of NVUs, comprising several cellular components, such as MGs, ACs, PCs, and ECs [6,7,8,27]. To study the microglial localization between ACs, PCs, and ECs, immunohistochemistry was performed on brain samples obtained from adult mice. Double immunohistochemical staining revealed the presence of Iba1^+^ MGs near glial fibrillary acidic protein (GFAP)^+^ ACs (Figure 1A–D), as well as alpha-smooth muscle actin (αSMA)^+^ PCs (Figure 1E–H), and CD31^+^ ECs (Figure 1I–L). It was also confirmed that Iba1^+^ MGs, especially those around blood vessels (Figure 1M,N), were present near GFAP^+^ ACs (Figure 1M,O), αSMA^+^ PCs (Figure 1M,P), and CD31^+^ ECs (Figure 1M,Q). These findings indicate that MGs are located near ACs, PCs, and ECs, serving in part as NVU components in the brain.

### 2.2. MG, AC, PC, and EC Expression Patterns in Pathological Brain Samples after Ischemic Stroke

The findings indicate that MGs take part in constituting the NVU, along with ACs, PCs, and ECs, in a healthy brain. However, under pathological conditions, such as ischemic stroke, NVUs could be disrupted, and certain AC, PC, and EC populations within and around ischemic areas could be altered, coupled with abundant microglial accumulation [16,17]. These results suggest that MG-surrounding EC, AC, and PC localization could change under pathological conditions.

**Figure 2:**
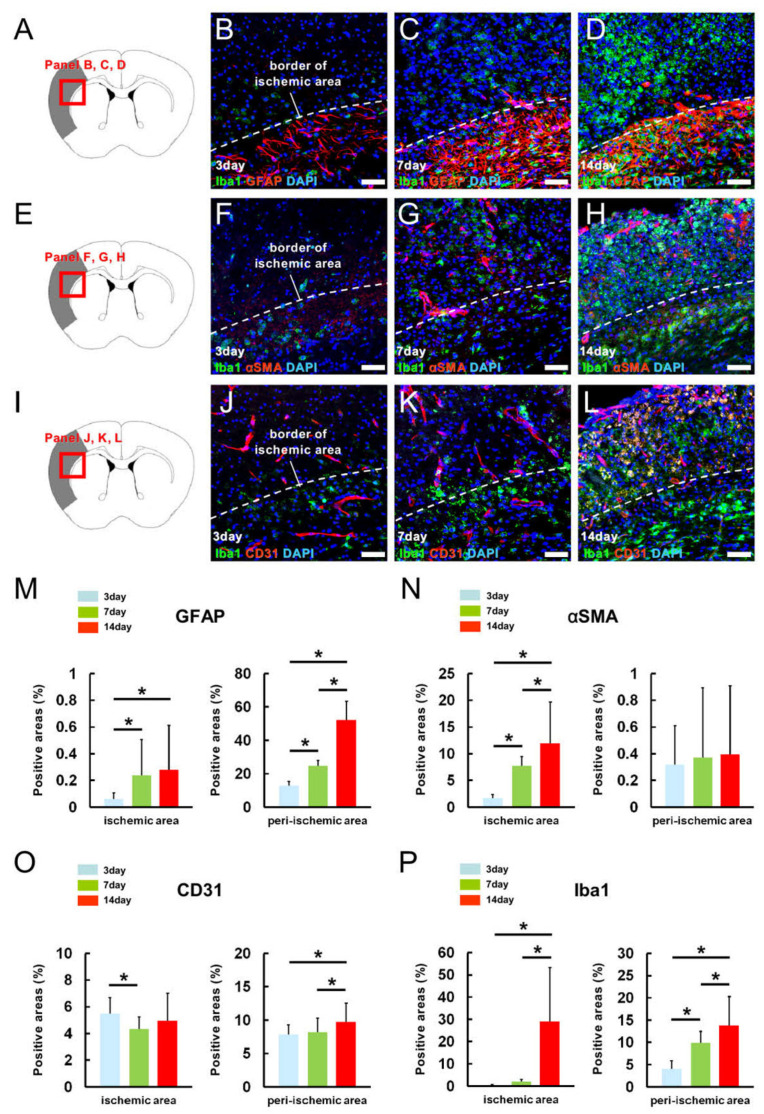
MG, AC, PC, and EC localizations and expression patterns in pathological brain samples. Double immunohistochemical staining (**A**–**L**) for Iba1/GFAP [Iba1 ((**B**–**D**): green); GFAP ((**B**–**D**): red); DAPI ((**B**–**D**): blue)], Iba1/αSMA [Iba1 ((**F**–**H**): green); αSMA ((**F**–**H**): red); DAPI ((**F**–**H**): blue)], and Iba1/CD31 [Iba1 ((**J**–**L**): green); CD31 ((**J**–**L**): red); DAPI ((**J**–**L**): blue)] in mice after ischemic stroke. Positive areas expressing GFAP (**M**), αSMA (**N**), CD31 (**O**), or Iba1 (**P**) were investigated within the ischemic and peri-ischemic areas 3, 7, and 14 days poststroke. Statistical analysis was performed using one-way ANOVA, followed by Bonferroni post-hoc tests ((**M**–**P**); 27 data points (3 points/section, 3 sections/brain, obtained from 3 mouse brains) were used for this analysis). * *p* < 0.05 among poststroke animals at 3, 7, and 14 days after MCAO. Scale bars: 50 µm (**B**–**D**,**F**–**H**,**J**–**L**). Abbreviations: AC, astrocyte; αSMA, alpha-smooth muscle actin; DAPI, 4’,6-diamidino-2-phenylindole; EC, endothelial cell; GFAP, glial fibrillary acidic protein; MCAO, middle cerebral artery occlusion; MG, microglial cell; PC, pericyte.

To address this, MG, AC, PC, and EC expression patterns were evaluated using a mouse stroke model. Immunohistochemistry staining at 3 (Figure 2A,B,M), 7 (Figure 2A,C,M), and 14 (Figure 2A,D,M) days poststroke revealed that GFAP^+^ areas were significantly increased at later time points within the ischemic and peri-ischemic areas. αSMA immunohistochemistry staining at 3 (Figure 2E,F,N), 7 (Figure 2E,G,N), and 14 (Figure 2E,H,N) days poststroke revealed that αSMA^+^ areas did not significantly differ in the peri-ischemic areas, while these were significantly increased at later time points within the ischemic areas. In contrast, CD31^+^ areas significantly decreased within the ischemic areas 7 days poststroke compared with those 3 days poststroke based on immunohistochemical staining at 3 (Figure 2I,J,O), 7 (Figure 2I,K,O), and 14 (Figure 2I,L,O) days poststroke. Although the CD31^+^ areas were significantly increased at the peri-ischemic areas at later time points (Figure 2O), it was only slightly different compared with those observed in ACs and PCs. Consistent with the results on GFAP^+^ ACs and αSMA^+^ PCs, Iba1^+^ areas were also prominently increased at later time points in ischemic and peri-ischemic areas based on immunohistochemistry staining at 3 (Figure 2B,F,J,P), 7 (Figure 2C,G,K,P), and 14 (Figure 2D,H,L,P) days poststroke. Several Iba1^+^ MGs were localized around GFAP^+^ ACs, αSMA^+^ PCs, and CD31^+^ ECs. These results indicate that MGs potentially establish cell–cell contacts with ACs, PCs, and ECs, even in pathological cases.

**Figure 3:**
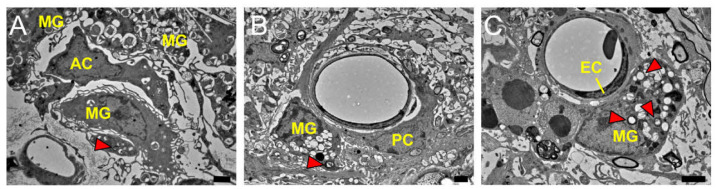
Electron microscopy at 14 days poststroke showed that MGs, which phagocytosed debris including damaged myelin (arrowhead), interacted with ACs (**A**), PCs (**B**), or ECs (**C**) in pathological brains. Scale bars: 2 µm (**A**–**C**). Abbreviations: AC, astrocyte; EC, endothelial cell; MG, microglial cell; PC, pericyte.

To confirm the cell–cell interactions between MGs and ACs, PCs, or ECs in pathological brains, electron microscopy was performed using brain samples obtained from poststroke mice. It was observed that MGs, which phagocytosed debris, including injured myelin, interacted with ACs (Figure 3A), PCs (Figure 3B), or ECs (Figure 3C) within ischemic areas. 

### 2.3. AC and PC Contact Increased the Number of MGs, while EC Contact Reduced Them

**Figure 4:**
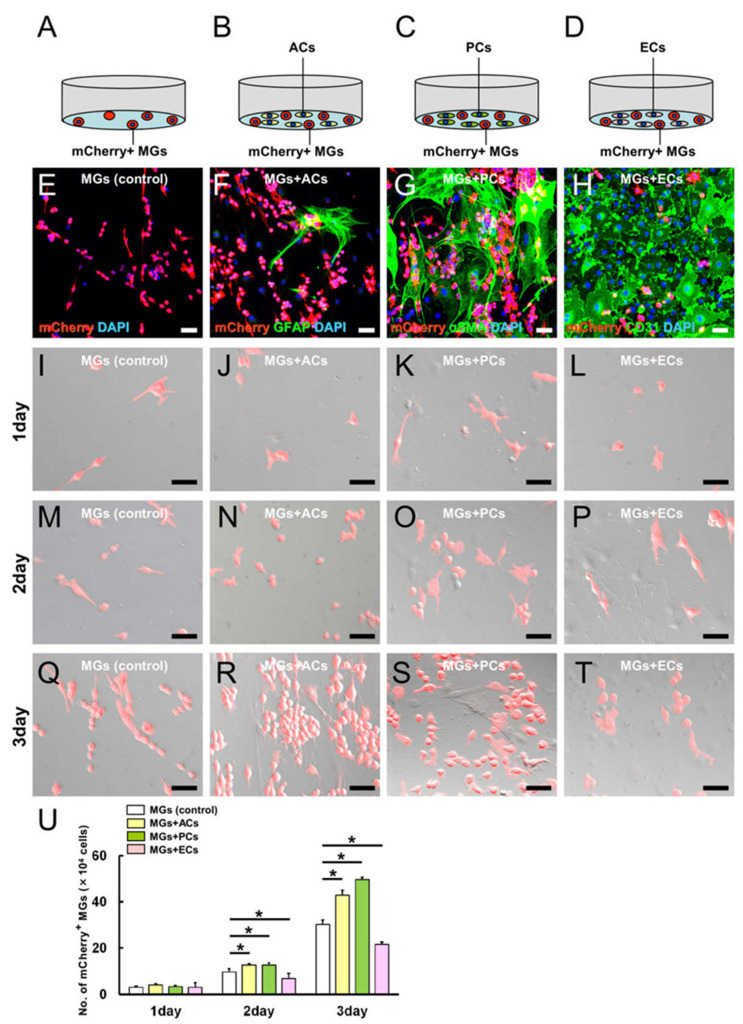
MGs alone (**A**) or those cocultured with ACs (**B**), PCs (**C**), or ECs (**D**) were incubated under direct cell–cell contact conditions. Immunohistochemical staining showed that mCherry^+^ MGs (**E**–**H**) were partly in contact with GFAP^+^ ACs (**F**), αSMA^+^ PCs (**G**), and CD31^+^ ECs (**H**). The number of mCherry^+^ MGs was evaluated in the MGs alone (**I**,**M**,**Q**), MGs + ACs (**J**,**N**,**R**), MGs + PCs (**K**,**O**,**S**), and MGs + ECs groups (**L**,**P**,**T**) at 1 (**I**–**L**), 2 (**M**–**P**), and 3 days (**Q**–**T**) after incubation. Compared with the control (MGs alone), the number of mCherry^+^ MGs did not significantly change in any group at 1 day after incubation (**I**–**L**,**U**). However, compared with the control (**M**,**Q**,**U**), the number of mCherry^+^ MGs significantly increased at 2 and 3 days after coincubation with ACs (**N**,**R**,**U**) or PCs (**O**,**S**,**U**). In contrast, the number of mCherry^+^ MGs significantly decreased 2 and 3 days after coincubation with ECs (**P**,**T**,**U**). Scale bars: 50 µm (**E**–**T**). Statistical analysis was performed using one-way ANOVA, followed by Bonferroni post-hoc tests (U; *n* = 4 for each group at 1, 2, or 3 day after incubation). * *p* < 0.05 among MGs alone, MGs + ACs, MGs + PCs, and MGs + ECs groups at the same time points after incubation. Abbreviations: AC, astrocyte; αSMA, alpha-smooth muscle actin; DAPI, 4’,6-diamidino-2-phenylindole; EC, endothelial cell; GFAP, glial fibrillary acidic protein; MG, microglial cell; PC, pericyte.

To investigate how ACs, PCs, and ECs could affect MGs, coculture experiments were performed under conditions that favored the establishment of cell–cell contacts. MGs were incubated alone or with ACs, PCs, or ECs (Figure 4A–D, respectively). mCherry^+^ MGs (5 × 10^4^ cells/well) were plated onto a six-well dish, and the same number (5 × 10^4^ cells/well) of ACs, PCs, or ECs was added to the culture 3 h later. On day 3 of incubation, the cells were fixed and subjected to immunohistochemical analysis, indicating that certain mCherry^+^ MGs (Figure 4E–H) established contacts with GFAP^+^ ACs (Figure 4F), αSMA^+^ PCs (Figure 4G), and CD31^+^ ECs (Figure 4H). Notably, the number of mCherry^+^ MGs cocultured with ACs or PCs increased, while those in EC cocultures decreased (Figure 4E–H).

To confirm this result, coculture experiments were performed under the same conditions as described above (Figure 4A–D), and mCherry^+^ MGs were counted 1, 2, and 3 days after the coincubation. Compared with the control (Figure 4I,M,Q,U), the number of mCherry^+^ MGs did not change in any group 1 day after coincubation (Figure 4J–L,U); it significantly increased 2 and 3 days after the coincubation with ACs (Figure 4N,R,U) or PCs (Figure 4O,S,U), but it significantly decreased 2 and 3 days when coincubated with ECs (Figure 4P,T,U). These findings show that coincubation with ACs and PCs increased the number of MGs, while ECs suppressed it.

### 2.4. ACs, PCs, and ECs Affect Microglial Numbers Dose-Dependently

**Figure 5:**
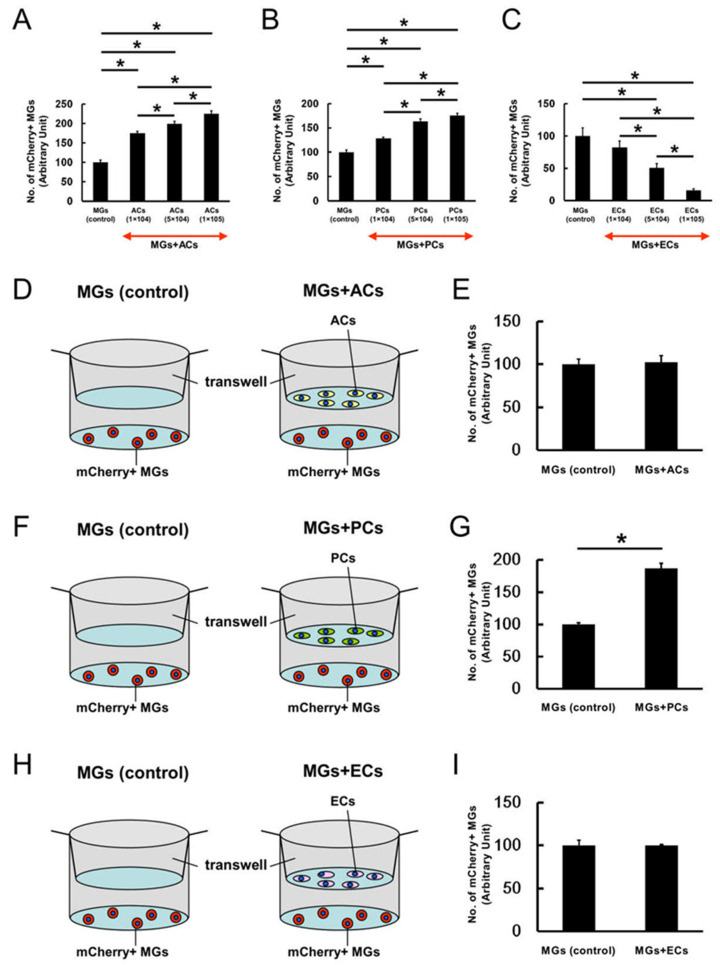
Whether ACs, PCs, or ECs affected microglial numbers dose-dependently was investigated under direct cell–cell contact conditions (**A**–**C**). The number of mCherry^+^ MGs significantly increased dose-dependently after coincubation with ACs (**A**) and PCs (**B**), but it was significantly decreased dose-dependently after coincubation with ECs (**C**). The same number of mCherry^+^ MGs and ACs (**D**), PCs (**F**), or ECs (**H**) were incubated under conditions that did not allow direct cell contacts. Compared with the control (mCherry^+^ MGs alone), ACs (**E**) and ECs (**I**) did not alter the number of mCherry^+^ MGs, while PCs significantly increased it (**G**). Statistical analysis was performed using a one-way ANOVA, followed by Bonferroni post-hoc tests ((**A**–**C**); *n* = 4 for each group) or the Student’s *t*-test ((**E**,**G**,**I**); *n* = 4 for each group). * *p* < 0.05 among the groups (**A**,**B**,**C**). * *p* < 0.05 between MGs alone (control) (**G**). Abbreviations: AC, astrocyte; EC, endothelial cell; MG, microglial cell; PC, pericyte.

Next, mCherry^+^ MGs (5 × 10^4^ cells/well) were coincubated with various numbers (1 × 10^4^, 5 × 10^4^, or 1 × 10^5^ cells/well) of ACs, PCs, or ECs in six-well dishes for 4 days. The number of mCherry^+^ MGs dose-dependently and significantly increased after coincubation with ACs (Figure 5A) and PCs (Figure 5B), but this was dose-dependently and significantly decreased when coincubated with ECs (Figure 5C).

Furthermore, whether direct cell–cell contact would be essential for exerting such effects was determined by incubating two cell types under conditions that do not support direct cell–cell contact. Briefly, mCherry^+^ MGs (2 × 10^4^ cells/well) were plated onto the bottom of 12-well dishes, followed by plating the same number (2 × 10^4^ cells/well) of ACs (Figure 5D), PCs (Figure 5F), or ECs (Figure 5H) into the wells of Transwell plates with 0.4 μm slits in the membrane. Then, the cells were incubated separately for 4 days. On day 4 of incubation, mCherry^+^ MGs were collected and counted. Compared with the control, the number of mCherry^+^ MGs did not significantly change in the groups coincubated with ACs (Figure 5E) and ECs (Figure 5I), but it significantly increased in the groups coincubated with PCs (Figure 5G). These data indicate that PCs increased MG numbers not only though direct cell–cell contacts but also under coculture conditions that disabled cell contacts.

### 2.5. Contact with ACs, PCs, or ECs Altered Microglial Phenotypes

The gene expression patterns in MGs were further investigated after incubating mCherry^+^ MGs alone or with ACs, PCs, or ECs for 3 days under conditions that enable the establishment of direct cell contacts. Then, mCherry^+^ MGs were sorted by FACS and subjected to microarray analysis.

Gene ontology (GO) analysis showed that MGs coincubated with ACs, PCs, and ECs exhibited an enrichment of cell fate-related genes, such as those involved in cell proliferation, adhesion, growth, death, regulation of apoptotic DNA fragmentation, adhesion, differentiation, or negative regulation of intrinsic apoptotic signaling pathways upon DNA damage (Table 1). These results indicate that establishing contacts with ACs, PCs, or ECs altered the fate of MGs.

### 2.6. Cell Cycle-Related Gene Expression Patterns in MGs upon AC, PC, or EC Contact

Cell cycle-related gene expression patterns were investigated. Our pathway analysis showed that 11 (Bub1b, Ccna1, Ccnd2, Cdc25a, Cdk2, Cdkn2a, E2f1, Mcm6, Rb1, Rbl1, and Trp53) and 2 (Ccnb3 and Ywhag) cell cycle-related genes were significantly up- or downregulated by more than two-fold, respectively, in MGs cocultured with ACs compared with those cultured alone (Supplementary Figure S1).

Moreover, nine (Bub1b, Ccnb3, Ccnd2, Ccnd3, Cdc25a, Cdkn1b, Gadd45a, Mcm6, and Rbl1) and six (Atr, Gsk3b, Mcm5, Rbl1, Skp2, and Trp53) cell cycle-related genes were found to be up- or downregulated by more than two-fold, respectively, in MGs cultured with PCs compared with those cultured alone (Supplementary Figure S2).

Finally, 13 (Bub1b, Ccna1, Ccnd2, Ccnd3, Cdc6, Cdh1, Cdkn1b, Cdkn2a, E2f1, Gadd45a, Mcm6, Mpeg1, and Rbl1) and 12 (Bub1, Ccnb1, Ccnb3, Ccnd3, Ccne1, Cdc6, Cdc25a, Chek1, Mcm3, Mcm5, Rbl1, and Trp53) cell cycle-related genes were up- or downregulated by more than two-fold, respectively, in MGs cultured with ECs compared with those cultured alone (Supplementary Figure S3). Supplementary Tables S1–S3 summarize the fold change values of the aforementioned genes between MGs cultured with ACs, PCs, and ECs, respectively, and alone (MGs with ACs, PCs, and ECs/MGs alone, respectively). These results suggest that ACs, PCs, or ECs potentially affect MG numbers partially through cell cycle regulation.

### 2.7. Apoptosis-Related Gene Expression Patterns in MGs upon AC, PC, or EC Contact

The expression patterns of apoptosis-related genes were investigated. Our pathway analysis identified 6 (Hrk, Igf2, Irf7, Nfkbie, Tnf, and Trp53) and 13 (Akt1, Diablo, Fasl, Gzmc, Ikbkb, Irf4, Irf6, Tnfsf10, Tnfrsf10b, Tnfrsf21, Tnfrsf25, Trp63, and Trp73) apoptosis-related genes that were significantly up- or downregulated by more than two-fold, respectively, in MGs cultured with ACs compared with those cultured alone (Supplementary Figure S4).

In MGs cultured with PCs compared with those cultured alone, 7 (Bcl2, Casp4, Casp6, Cflar, Igf1r, Ikbkg, and Trp63) and 15 (Birc2, Casp3, Gzmc, Hells, Igf1, Ikbkb, Irf4, Mapk10, Nfkbia, Pik3r1, Tnf, Tnfsf10, Traf1, Trp53, and Trp63) apoptosis-related genes were up- or downregulated by more than two-fold, respectively (Supplementary Figure S5).

Finally, 14 genes upregulated by more than two-fold (Bcl2, Bok, Casp4, Casp6, Cflar, Hrk, Igf2, Ikbkb, Ikbkg, Irf7, Nfkbie, Tnfsf10, Tnfrsf10b, and Trp63) and 14 downregulated (Akt1, Fasl, Gzmc, Hells, Ikbkb, Irf6, Mapk10, Nfkbia, Tnf, Tnfsf10, Tnfrsf21, Traf1, Trp53, and Trp63) apoptosis-related genes were found in MGs cultured with ECs compared with those cultured alone (Supplementary Figure S6). Supplementary Tables S4–S6 summarize the fold change values of the aforementioned genes between MGs cultured with ACs, PCs, and ECs, respectively, and alone (MGs with ACs, PCs, and ECs/MGs alone, respectively). These results suggest that ACs, PCs, or ECs might influence the number of MGs in part by cell death regulation.

### 2.8. M1/M2 Marker Expression Patterns in MGs upon AC, PC, or EC Contact

**Figure 6:**
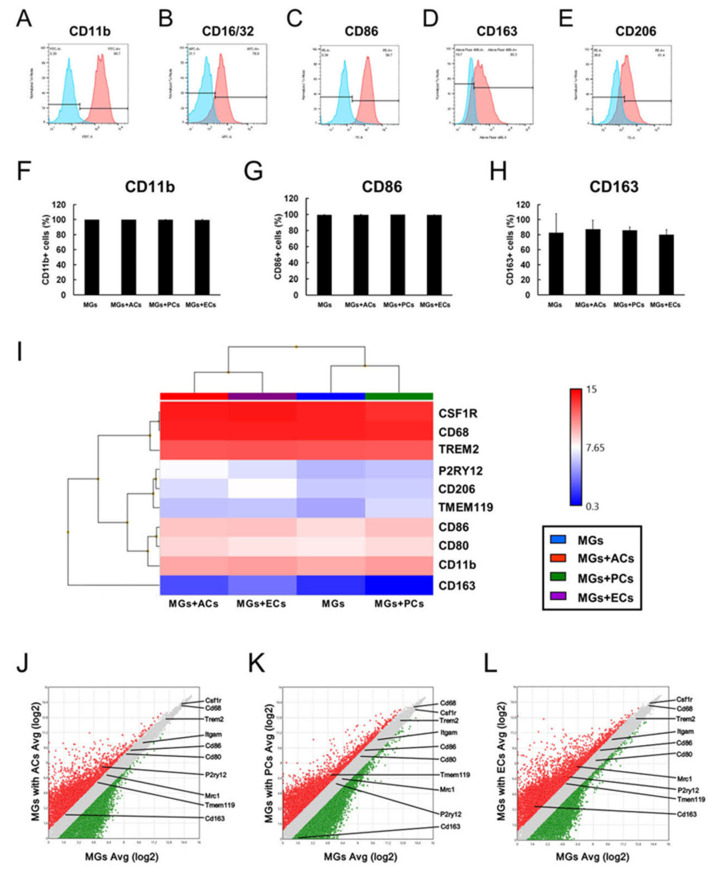
MG markers investigated by FACS analysis (**A**–**H**). MGs expressed various markers: CD11b (**A**), CD16/32 (**B**), CD86 (**C**), CD163 (**D**), and CD206 (**E**). FACS analysis also showed that the presence of ACs, PCs, or ECs did not affect the CD11b+ (**F**), CD86+ (**G**), and CD163+ cell populations (**H**) compared with those in cultures of MGs alone (**F**–**H**). Heat mapping analysis of MG-related gene expression patterns (**I**). Scatter plot analysis of MG-related genes between MGs cultured alone and MGs coincubated with ACs (**J**), PCs (**K**), or ECs (**L**). Statistical analysis was performed using one-way ANOVA, followed by Bonferroni post-hoc tests ((**F**–**H**); *n* = 3 for each group). Abbreviations: AC, astrocyte; EC, endothelial cell; MG, microglial cell; PC, pericyte.

MGs display two phenotypes: M1 and M2. M1 and M2 cells exert cytotoxic and cytoprotective effects, respectively [15]. Therefore, whether direct contact with ACs, PCs, or ECs would affect the M1/M2 MG phenotypes was investigated. First, MG cell surface markers were analyzed before the coculture experiments. FACS analysis showed that MGs expressed conventional (CD11b (99.7%, Figure 6A)), M1 (CD16/32 (78.9%, Figure 6B) and CD86 (99.7%, Figure 6C)), and M2 (CD163 (80.3%, Figure 6D) and CD206 (61.4%, Figure 6E)) type markers.

Then, the M1/M2 expression patterns of MGs were investigated after being cultured with ACs, PCs, or ECs under conditions that enable direct cell–cell contacts, incubating mCherry^+^ MGs alone with the same number of ACs, PCs, or ECs for three days. mCherry^+^ MGs were analyzed by FACS, which revealed no significant difference in CD11b^+^ (Figure 6F), CD86^+^ (Figure 6G), and CD163^+^ (Figure 6H) cell populations between the control and AC-, PC-, or EC-cocultured MGs. Heat mapping analysis was also performed, which also showed that the conventional (CD11b (ITGAM), CSF1R, TREM2, TMEM119, and P2RY12), M1 (CD68, CD80, and CD86), and M2 (CD163 and CD206 (MRC1)) microglial marker gene expression patterns did not markedly differ among these groups (Figure 6I).

Supplementary Tables S7–S9 summarize the fold change values of the aforementioned genes between MGs cultured with ACs, PCs, and ECs, respectively, and alone (MGs with ACs, PCS, and ECs/MGs alone, respectively). Scatter plot analysis shows the distribution of these genes between MGs cultured alone and those cocultured with ACs, PCs, or ECs (Figure 6J–L, respectively).

The results showed that the gene expression levels of these markers were all restricted within two-fold in MGs cocultured with ACs (Figure 6J) and highly restricted within two-fold in MGs cocultured with PCs (Figure 6K) and ECs (Figure 6L) compared with those in MGs cultured alone (MGs with ACs, PCs, and ECs/MGs alone, respectively). Therefore, we could conclude that ACs, PCs, or ECs exert a very limited effect on M1/M2 type microglial phenotypic alternations after a cell–cell contact.

### 2.9. Gja1 Involvement in Microglial Regulation upon AC, PC, or EC Contact

Key factors that might influence the number of MGs after a cell–cell contact were further investigated by comparing the gene expression patterns of MGs cocultured with ACs, PCs, and ECs with those of MGs cultured alone (MGs with ACs, PCs, and ECs/MGs alone, respectively); the top 10 highly expressed genes under each condition are shown in Table 2, Table 3 and Table 4, respectively.

**Figure 7:**
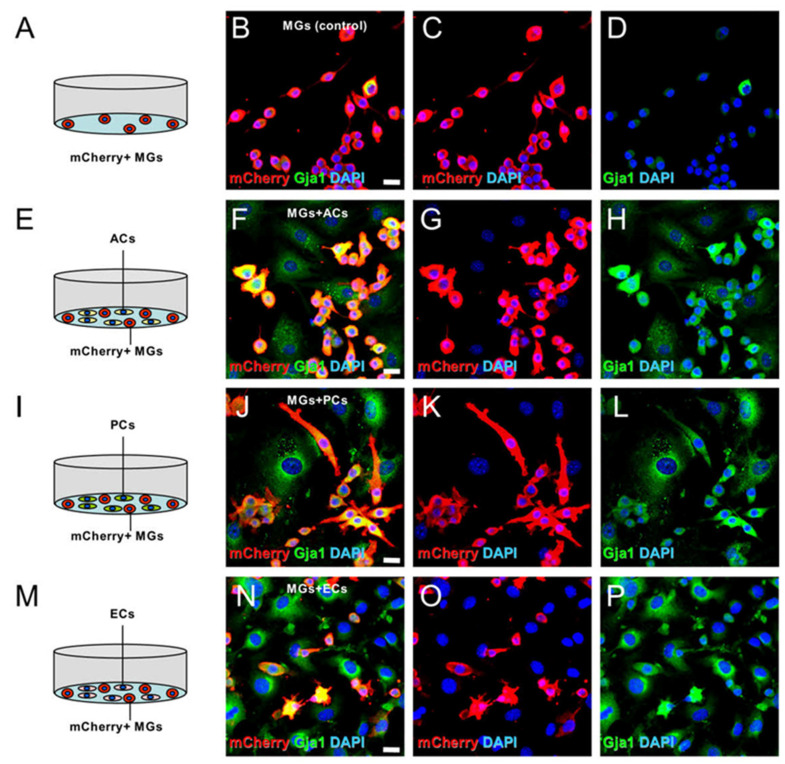
Immunohistochemical staining for Gja1 expression in MGs cultured alone (**A**–**D**) or with ACs (**E**–**H**), PCs (**I**–**L**), or ECs (**M**–**P**) (mCherry (**B**,**C**,**F**,**G**,**J**,**K**,**N**,**O**: red); Gja1 (**B**,**D**,**F**,**H**,**J**,**L**,**N**,**P**: green); DAPI (**B**–**D**,**F**–**H**,**J**–**L**,**N**–**P**: blue)). The results represent triplicates. Scale bars: 20 µm (**B**,**F**,**J**,**N**). Abbreviations: AC, astrocyte; DAPI, 4’,6-diamidino-2-phenylindole; EC, endothelial cell; Gja1, gap junction protein, alpha 1; MG, microglial cell; PC, pericyte.

Gap junction protein alpha 1 (Gja1, also known as connexin 43), Cnn3, and Fscn1 were found to be commonly upregulated in MGs upon cell–cell contact with ACs, PCs, or ECs. These results were supported by previous studies, describing that stimulated MGs had upregulated Gja1 [28], Cnn3 [29], and Fscn1 [30] expressions. Among these genes, Gja1 was prominently upregulated in MGs after a cell–cell contact with ACs. Consistent with previous studies [28,31,32], immunohistochemical staining proved that Gja1 was present in certain MGs not cocultured with other cell types (Figure 7A–D). However, Gja1 was strongly expressed in mCherry^+^ MGs coincubated with ACs (Figure 7E–H), PCs (Figure 7I–L), or ECs (Figure 7M–P). Gja1 was also expressed in mCherry^−^ cells, including ACs (Figure 7E–H), PCs (Figure 7I–L), or ECs (Figure 7M–P). These results support the previous reports that Gja1 was strongly expressed in activated MGs [28] and ACs, PCs, and ECs [33,34].

**Figure 8:**
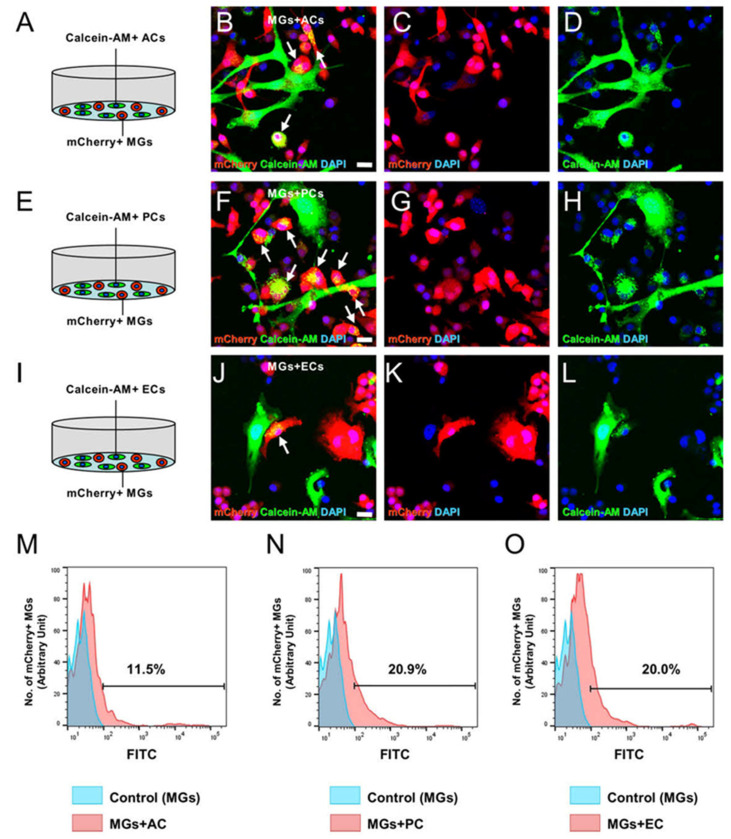
Transition of calcein-AM from ACs, PCs, or ECs into MGs after coincubation. Immunofluorescent examination for calcein-AM expression in MGs cocultured with ACs (**A**–**D**), PCs (**E**–**H**), or ECs (**I**–**L**) [mCherry ((**B**,**C**,**F**,**G**,**J**,**K**): red); calcein-AM ((**B**,**D**,**F**,**H**,**J**,**L**): green); DAPI ((**B**–**D**,**F**–**H**,**J**–**L**): blue)]. Immunofluorescent analysis showed that calcein-AM was detectable in mCherry^+^ cells cocultured with ACs ((**B**), arrows), PCs ((**F**), arrows), or ECs ((**J**), arrows). The transition of calcein-AM from ACs (**M**), PCs (**N**), or ECs (**O**) to MGs was observed by FACS analysis. The control indicates the samples of MGs alone. The results represent triplicates. Scale bars: 20 µm (**B**,**F**,**J**). Abbreviations: AC, astrocyte; DAPI, 4’,6-diamidino-2-phenylindole; EC, endothelial cell; MG, microglial cell; PC, pericyte.

To obtain a direct evidence that Gja1 expression in MGs was regulated by ACs, PCs, or ECs via intercellular interactions, the transition of calcein-AM was investigated between contacted cells. ACs, PCs, or ECs prelabeled with calcein-AM were cocultured with mCherry^+^ MGs. Then, these were fixed and subjected to immunofluorescent examination. The results showed that although calcein-AM was not observed in control mCherry^+^ MGs, calcein-AM was observed in mCherry^+^ MGs cocultured with ACs (Figure 8A–D), PCs (Figure 8E–H), or ECs (Figure 8I–L). FACS analysis also showed that calcein-AM was detected as FITC signaling in mCherry^+^ cells cocultured with ACs (11.5%; Figure 8M), PCs (20.9%; Figure 8N), or ECs (20.0%; Figure 8O).

**Figure 9:**
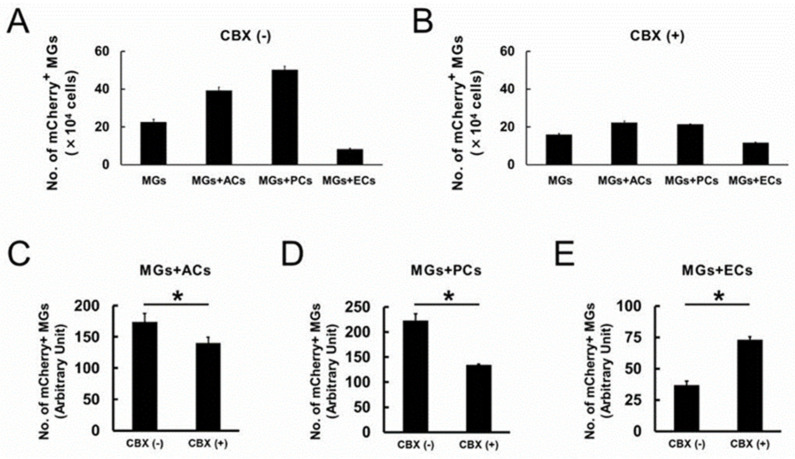
Number of mCherry^+^ MGs after incubation without CBX (**A**) or with CBX (**B**) ((**A**,**B**); *n* = 3 for each group). The number of mCherry^+^ MGs was analyzed for MGs coincubated with ACs (**C**), PCs (**D**), or ECs (**E**) between the CBX(−) and CBX(+) groups. The results show that the presence of CBX significantly reduced the number of MGs coincubated with ACs (**C**) or PCs (**D**), but it significantly increased that of MGs coincubated with ECs (**E**). Statistical analysis was performed using the Student’s *t*-test ((**C**–**E**); *n* = 3 for each group). * *p* < 0.05 between the CBX (−) and CBX (+) groups (**C**–**E**). Abbreviations: AC, astrocyte; CBX, carbenoxolone; EC, endothelial cell; MG, microglial cell; PC, pericyte.

Finally, to investigate how Gja1 functions in MGs coincubated with ACs, PCs, or ECs, the coculture medium was supplemented with carbenoxolone (CBX), a Gja1 inhibitor [35]. Briefly, mCherry^+^ MGs (2 × 10^4^ cells/well) were plated onto a 12-well dish, and the same number (2 × 10^4^ cells/well) of ACs, PCs, or ECs were added into the wells 3 h later. One day after incubation, the coculture medium was supplemented with CBX (50 µM). Then, the cells were incubated for 3 more days and the number of mCherry^+^ MGs was evaluated by FACS analysis. These results show that the number of MGs cocultured with ACs (39.28 ± 1.75 and 22.31 ± 0.88 cells/well under CBX (−) and CBX (+) conditions, respectively) or PCs (50.31 ± 1.80 and 21.52 ± 0.08 cells/well under CBX (−) and CBX (+) conditions, respectively) was smaller under CBX (+) compared with those under CBX (−) conditions. In contrast, the number of MGs cocultured with ECs (8.32 ± 0.44 and 11.67 ± 0.22 cells/well under CBX (−) and CBX (+) conditions, respectively) was larger under CBX (+) compared with that under CBX (−) conditions (Figure 9A,B). 

Then, the number of MGs cultured alone under CBX (−) (Figure 9A) and CBX (+) conditions (Figure 9B) was compared. CBX suppressed the number of MGs (22.57 ± 1.53 and 15.95 ± 0.62 cells/well under CBX (−) and CBX (+) conditions, respectively) (Figure 9A,B).

Therefore, the mean number of MGs obtained from CBX (−) or CBX (+) cultures was set as a standard (100) point on two extremities of the Y-axis, respectively. The values of MGs cocultured with ACs, PCs, or ECs under CBX (−) or CBX (+) conditions were converted after dividing them by this standard. Then, the number of MGs cultured with ACs, PCs, and ECs under CBX (−) was compared with that under CBX (+) conditions. The results showed that CBX-mediated Gja1 inhibition significantly reduced the number of MGs cocultured with AC and PC (Figure 9C,D), indicating that ACs and PCs contributed to the increase in microglial number via Gja1. However, a significant increase in the number of MGs was observed upon the same treatment in EC cocultures (Figure 9E), indicating that ECs contributed to reducing the number of MGs via Gja1. These findings indicate that the function of Gja1 differs depending on the contacted cell types. The findings support recent studies claiming that Gja1-derived effects differ among the contacted cell types [36].

## 3. Discussion

Although MGs are present in various brain regions, an increasing body of evidence demonstrates that they are present in some parts of NVU, which is mainly composed of ECs, ACs, and PCs [6,7,8]. MGs are pivotal in maintaining NVU function by regulating the BBB permeability and CBF under both physiological and pathological conditions [9,10,11,12,14,15]. However, how ECs, ACs, and PCs affect MGs has been scarcely investigated. In this study, we described how NVU-forming cells, including ECs, ACs, and PCs, exert diverse effects on MGs. In addition, our coculture experiments showed that ECs reduced the number of MGs, while ACs and PCs increased it. Therefore, these NVU-composing cells might balance its number in the brain.

MGs could be classified into M1 and M2 subtypes. M1 are proinflammatory, while M2 are anti-inflammatory MGs, exerting cytotoxic and cytoprotective effects on the brain (e.g., BBB disruption and protection), respectively [15]. Notably, several cytokines (e.g., tumor necrosis factor (Tnf) or interferon-γ (IFN-γ)) can shift MGs toward M1 polarization, while certain interleukin (IL) types, such as IL-4 and IL-12, could induce M2 polarization [37]. Cell–cell contact between ACs and MGs could also contribute to triggering M2 polarization in the latter [38]. However, this study showed that direct cell–cell contacts between MGs and ACs, PCs, or ECs did not or rarely altered the M1/M2 phenotypes. Although the reason for this discrepancy with a previous study [38] is unknown, several differences in the execution of coculture experiments (e.g., duration of coincubation) might account for it.

In the present study, both AC and PC significantly increased in numbers, especially around and within the ischemic areas, respectively, similar to MG numbers. Under physiological conditions, MGs are present in the brain in a quiescent state. However, upon brain injuries [39], MGs became active and highly proliferate due to several factors (e.g., IL-1β, IL-4, Tnf-α, and GM-CSF) [38,40]. Although a previous study showed that ACs and/or PCs secrete some of these factors (e.g., Tnf-α and GM-CSF) [41], they also triggered an increase in MG number upon direct contact in our coculture experiments. Although the exact underlying effector mechanisms remain unclear, a previous study described that ACs promoted microglial proliferation in coculture experiments [38]. Our study also showed that direct cell–cell contacts between MGs and ACs, PCs, or ECs regulate cell cycle- or apoptosis-related gene expressions in MGs. GO analysis also showed that several cell fate-related genes, including cell proliferation, cell death, and apoptosis, were enriched in MGs after a cell–cell contact with ACs, PCs, or ECs. Therefore, such factors might partially affect microglial numbers upon cell contact.

In this study, Cnn3 and Fscn1 were prominently upregulated in MGs cocultured with ACs, PCs, or ECs, indicating that microglial phenotypes were altered upon being in direct cell contact with these cells. Although its precise role in MGs remains unclear, Cnn3 was reportedly highly expressed de novo in activated MGs upon IFN-γ stimulation [29]. Moreover, Fscn1 was highly expressed in MGs after a spinal cord injury and regulated microglial migration [30].

In addition to Cnn3 and Fscn1, Gja1 was also significantly upregulated in MGs cocultured with ACs and PCs. Gja1 is a gap junction component that enables intercellular communication and regulates proliferation [42]. Although Gja1 plays an important role in brain homeostasis and brain diseases, its precise roles in specific cell types remain unclear [43,44]. Notably, while Gja1 was found to be involved in increasing AC and PC numbers, it also contributed to reducing EC numbers, indicating that ACs and PCs positively regulated the number of MGs via Gja1, while ECs reduced it. These results support recent studies that Gja1-derived effects differ among the contacted cell types [36].

PCs, key NVU components, together with ACs and ECs, are pivotal in BBB maintenance [9,10,11]. PC ablation increases the BBB permeability and induces BBB disruption [9,45]. PC depletion also causes inflammatory responses and perivascular MG infiltration [46]. These findings suggest that PCs play an important role in regulating inflammatory cell fates under inflammatory conditions, such as ischemic stroke. Consistent with a previous study [17], our results showed that the number of MGs increased within ischemic areas after the onset of ischemic stroke in parallel with pericyte numbers. Furthermore, PCs also increased MG numbers upon direct cell contact and under coculture conditions that disabled cell contacts. How PCs affect MGs remains unclear. However, they  secrete various factors (e.g., cytokines and chemokines) that regulate microglial phenotypes [41,47]. Therefore, cell contacts and PC-derived soluble factors might affect the fate of MGs, thereby increasing its numbers.

In a healthy brain, ECs constitute the BBB formed by interendothelial junctions, including tight and adherens junctions. EC-cell junctions maintain the function of NVU by regulating intercellular junctions [48]. In this study, ECs reduced MG numbers upon direct cell contact. Therefore, under physiological conditions, ECs might negatively regulate MG numbers. Like PCs, ECs are resistant to hypoxia and could survive even after an ischemic stroke [17,20,21,22,23]; this was supported by this study because certain CD31^+^ ECs were present in ischemic areas. However, under pathological conditions, endothelial intercellular junctions are loosened, and injured ECs cause BBB dysfunction [49,50]. Such functionally damaged ECs might lose their proper MG regulatory ability, thereby increasing the number of MG in a pathological brain.

It is unclear where damaged ECs could reconstruct the BBB after brain injuries. However, our recent study showed that the endothelial number around ischemic areas increased during chronic periods compared with that during acute periods [17]. In addition, using genetic mapping for the EC marker VE-cadherin, we demonstrated that VE-cadherin expression increased both the promoter and protein levels in ischemic areas after stroke [20]. Similarly, the present study showed that CD31^+^ areas were significantly increased in peristroke areas at later time points. However, there was a slight increase in CD31^+^ areas 14 days poststroke. ACs and PCs, rather than ECs, might mainly regulate the number of MGs 14 days poststroke. However, ECs could suppress microglial numbers as ECs prominently increase by developing new blood vessels at later time points [17,22]. This hypothesis probably explains why microglial numbers peaked at acute stroke periods but decreased later on [51].

Our study has certain limitations. For example, microglial traits after cell contacts between MGs and ACs, PCs, or ECs might differ in coculture experiments under different conditions (e.g., hypoxia, glucose deprivation, or chemical stimulation). Whether Gja1 plays a significant role in microglial regulation upon cell–cell contact in vivo between MGs and ACs, PCs, or ECs remains unclear. However, in our preliminary study, Gja1 expression was observed in both nonischemic and ischemic areas, suggesting that Gja1 plays an important role in physiological and pathological brains. Furthermore, obtaining certain cell types (e.g., MGs and ECs) was difficult as primary cultures. Thus, established cell lines were used in this study. Although the precise traits of MG cell lines used in this study remain unclear, heat mapping analysis showed that the expression level of some typical markers (e.g., TMEM119 and P2RY12) was not prominently higher than expected. In addition, heat mapping and FACS analysis showed that these cells predominantly expressed M1 markers (CD68, CD80, and CD86) compared with M2 markers (CD163 and CD206). Thus, the MG cell lines used in this study may be closer to the features of M1 macrophages. Therefore, these issues might have affected the results of the coculture experiments, so these aspects should be addressed in future studies.

## 4. Materials and Methods

### 4.1. Animals and Brain Sample Preparation

The experimental procedures were approved by the Animal Care Committee of Hyogo Medical University (License number: 20-030, 22-052). We used 8–14-week-old adult mice (CB-17/Icr-+/+Jcl mice (CB-17 mice; CLEA Japan Inc., Tokyo, Japan)). Permanent focal cerebral ischemia was produced in mice by ligation and interruption of the distal portion of the left middle cerebral artery (MCA), as described previously [17,18,20,22,23,52,53]. Briefly, under general anesthesia by isoflurane inhalation, a burr hole was made in the skull using a drill (H021 Minimo; Minitor Co., Ltd., Tokyo, Japan). After opening the dura mater, MCA occlusion (MCAO) was made by electrocoagulation, followed by disconnection of the left MCA. Animals had access to food ad libitum, and efforts were made to minimize the number of animals used and their suffering. Before collecting healthy and ischemic brain samples (*n* = 3 for each sample), a mixture of medetomidine (0.3 mg/kg), midazolam (4 mg/kg), and butorphanol (5 mg/kg) were administered intraperitoneally to the mice (10 mL/kg) [54], and they were transcardially perfused with 4% paraformaldehyde (PFA), as described previously [17,18,20,22,23,52,53]. Whole brains were removed and subjected to postfixation with 4% PFA for 24 h. Then, the fixed brains were cryoprotected in 30% sucrose, frozen at −80 °C, and cut into 16 μm sections using a cryostat for immunohistochemistry.

### 4.2. Immunohistochemistry

Brain sections (20 μm thick) were subjected to immunohistochemistry using primary antibodies against Iba1 (1:500, goat, Abcam, Cambridge, UK), GFAP (1:500, rabbit, Abcam),αSMA (1:100, rabbit, Abcam), and CD31 (1:100, rat, BD Pharmingen, San Diego, CA, USA). The bound primary antibodies were visualized using Alexa Fluor 488-, 555-, 594-, or 647-conjugated secondary antibodies (1:500, Molecular Probes, Eugene, OR, USA). Cell nuclei were stained with 4’,6-diamidino-2-phenylindole (DAPI; 1:500, Kirkegaard & Perry Laboratories, Inc., Gaithersburg, MD, USA). Images were captured using a laser scanning microscope (LSM780, Carl Zeiss AG, Oberkochen, Germany; FV3000RS, Olympus, Tokyo, Japan).

Using coronal brain sections obtained from the same region for each animal, 27 data points (three points/section, three sections/brain, taken from three brains) from “ischemic” and “peri-ischemic” areas were analyzed using the Image J software and subjected to semiquantitative analysis, as previously described [17,20,22]. “Ischemic” and “peri-ischemic” areas were defined as those within the border of the poststroke area and those within its 100 μm border, respectively [17,20,22].

### 4.3. Electron Microscope

Electron microscopic analysis was performed [52,55]. Under deep anesthesia, the mice were perfused with a phosphate buffer solution containing 2% PFA and 2% glutaraldehyde at pH 7.4. Then, tissues were collected from the ischemic brain, and these were cut into 200 μm-thick slices using a vibratome. These sections were subjected to postfixation with 1% osmium tetroxide. During the procedures with a graded alcohol, the sections were stained with uranyl acetate solution. The samples were embedded in epoxy resin and cut into 1 μm-thick slices using an ultramicrotome, followed by staining with toluidine blue. For electron microscopy, 100 nm ultrathin sections were cut and arrayed on formvar-coated grid. The ultrathin sections were stained with a lead citrate solution, and the grids were observed under an electron microscope.

### 4.4. Cell Cultures

Commercially available mouse brain MGs (#SCC134, EMD Millipore Corporation, Temecula, CA, USA), ACs (#M1800, ScienCell Research Laboratories, Carlsbad, CA, USA), PCs (#M1200, ScienCell Research Laboratories), and ECs (CRL-2299, ATCC, Manassas, VA, USA) were obtained and maintained in a medium, following the manufacturer’s protocol. MGs were transduced with an mCherry-expressing lentivirus for labeling [22,23,56,57]. Then, mCherry^+^ MGs were subjected to fluorescence-activated cell sorting (FACS).

To investigate how ACs, PCs, or ECs affect MGs, mCherry^+^ MGs were plated into dishes (Thermo Fisher Scientific, Rochester, NY, USA). After coincubation with ACs, PCs, or ECs, the cells were fixed and subjected to immunohistochemistry using primary antibodies against mCherry (1:500, rabbit, Abcam [ab167453]; 1:1000, chicken, Abcam [ab205402]), GFAP (1:500, rabbit, Abcam), αSMA (1:100, mouse, EMD Millipore Corporation), CD31 (1:50, rat, BD Pharmingen), and Gja1 (1:100, rabbit, Proteintech, Rosemont, IL, USA). Bound primary antibodies were visualized using Alexa Fluor 488- and 555-conjugated secondary antibodies (1:500, Molecular Probes). Cell nuclei were stained with DAPI (1:500, Kirkegaard & Perry Laboratories, Inc.). Images were captured using a confocal laser scanning microscope (LSM780; Carl Zeiss AG).

ACs, PCs, or ECs were labeled with calcein-AM (2 μM, Dojindo laboratories, Kumamoto, Japan) for 20 min. Then, calcein-AM-labeled cell types were cocultured with mCherry^+^ MGs for 2 days. To investigate the transfer of calcein-AM from ACs, PCs, or ECs into MGs, the samples were subjected to histological examination or FACS analysis. In another set of experiments, CBX (Sigma, St. Louis, MO, USA) was added to the cocultures. 

### 4.5. Flow Cytometric Analysis

MGs were subjected to FACS analysis using PE-conjugated antibodies against CD11b (Thermo Fisher Scientific, Waltham, MA, USA), CD16/32 (Thermo Fisher Scientific), CD86 (Thermo Fisher Scientific), CD163 (Abcam), and CD206 (Thermo Fisher Scientific) for cell surface marker analysis in a FACS device (BD LSRFortessa™X-20, BD Pharmingen), as described previously [22]. In another set of experiments, mCherry^+^ MGs were collected by FACS after coincubation with ACs, PCs, or ECs, and the number of MGs was evaluated. To evaluate the transfer of calcein-AM from ACs, PCs, or ECs into MGs, mCherry^+^ MGs were collected by FACS, and the population of FITC-positive cells was investigated. In certain experiments, FACS-collected mCherry^+^ MGs were subjected to microarray analysis.

### 4.6. Microarray Analysis

Total RNA was isolated from controlled mCherry^+^ MGs and mCherry^+^ MGs coincubated with ACs, PCs, or ECs using an RNeasy Micro Kit (Qiagen, Hilden, Germany). Then, the samples (*n* = 1, for each group) were subjected to microarray analysis, as described previously [53,58]. The data were analyzed using an Affymetrix transcriptome analysis console. GO analysis was performed using the “GO enrichment analysis and visualization tool (Gorilla)” (http://cbl-gorilla.cs.technion.ac.il/), as described previously [59]. Pathway analysis was performed using WikiPathways (https://www.wikipathways.org/index.php/WikiPathways), as described [60,61].

### 4.7. Statistical Analysis

The data are presented as mean ± standard deviation. Statistical comparisons between multiple groups were done using one-way analysis of variance, followed by Bonferroni post-hoc tests. Student’s *t*-test was also performed for comparisons. *p*-values < 0.05 were considered statistically significant.

## 5. Conclusions

ACs and PCs increased the number of MGs, while ECs reduced it upon cell–cell contact. Although the precise underlying effector mechanism remains unclear, Gja1 was partially involved in regulating microglial proliferation. In addition, the gene expression patterns in MGs differed depending on the contacted cell types. Therefore, when considering inflammation regulation in the brain under physiological and pathological conditions, we should note that MG traits in the brain might vary depending on MG-surrounding cell types, such as ACs, PCs, or ECs.

## Figures and Tables

**Table 1:** GO analysis enrichment scores categorizing cell fate-related genes.

Categories	GO Term	Enrichment Score by “MGs with ACs/MGs Alone” Analysis	Enrichment Score by “MGs with PCs/MGs Alone” Analysis	Enrichment Score by “MGs with ECs/MGs Alone” Analysis
cell proliferation	GO:0008283	2.93	ND	ND
cell adhesion	GO:0007155	1.55	ND	2.81
regulation of cell proliferation	GO:0042127	1.77	ND	2.52
regulation of cell growth	GO:0001558	2.19	ND	ND
regulation of growth	GO:0040008	2.02	ND	ND
regulation of cell death	GO:0010941	1.74	ND	3.06
regulation of apoptotic DNA fragmentation	GO:1902510	ND	153.2	ND
regulation of cell adhesion	GO:0030155	1.95	ND	1.59
regulation of cell differentiation	GO:0045595	1.6	ND	1.79
positive regulation of apoptotic DNA fragmentation	GO:1902512	ND	229.8	2907.5
positive regulation of cell adhesion	GO:0045785	4.0	ND	ND
positive regulation of cell differentiation	GO:0045597	1.84	ND	ND
negative regulation of intrinsic apoptotic signaling pathway in response to DNA damage	GO:1902230	10.9	ND	ND
negative regulation of cell differentiation	GO:0045596	1.62	ND	ND

Abbreviation, ND; non-detectable.

**Table 2:** List of top 10 highly expressed genes in MGs with ACs compared with MGs alone.

Gene Symbol	Gene Name	ID	Fold Change (MGs with ACs/MGs Alone)
Gja1	gap junction protein, alpha 1	1415800_at	1130.13
Cnn3	calponin 3, acidic	1436759_x_at	565.68
Gja1	gap junction protein, alpha 1	1438945_x_at	378.16
Fstl1	follistatin-like 1	1416221_at	374.04
Serpinh1	serine (or cysteine) peptidase inhibitor, clade H, member 1	1450843_a_at	248.39
Gpr27	G protein-coupled receptor 27	1434848_at	240.42
Bgn	biglycan	1437889_x_at	215.44
Fstl1	follistatin-like 1	1448259_at	199.03
Fscn1	fascin homolog 1, actin bundling protein (Strongylocentrotus purpuratus)	1416514_a_at	183.35
Gja1	gap junction protein, alpha 1	1437992_x_at	159.71

**Table 3:** List of top 10 highly expressed genes in MGs with PCs compared with MGs alone.

Gene Symbol	Gene Name	ID	Fold Change (MGs with ACs/MGs Alone)
Csprs; Gm15433; Gm2666; Gm7609; LOC100041903; LOC100503923; LOC101055758	component of Sp100-rs; predicted pseudogene 15433; predicted gene 2666; predicted pseudogene 7609; proteinase-activated receptor 1-like	1435792_at	209.5
Serpinb2	serin (or cysteine) peptidase inhibitor, clade B, member 2	1419082_at	150.18
Cnn3	calponin 3, acidic	1436759_x_at	115.21
Igfbp3	insulin-like growth factor binding protein 3	1423062_at	97.18
Fstl1	follistatin-like 1	1416221_at	88.44
Fscn1	fascin homolog 1, actin bundling protein (Strongylocentrotus purpuratus)	1416514_a_at	72.77
Serpinh1	serin (or cysteine) peptidase inhibitor, clade H, member 1	1450843_a_at	65.99
Gja1	gap junction protein, alpha 1	1415800_at	59.89
Cxd5	chemokine (C-X-C motif) ligand 5	1419728_at	56.8
Col6a1	collagen, type VI, alpha 1	1448590_at	47.36

**Table 4:** List of top 10 highly expressed genes in MGs with ECs compared with MGs alone.

Gene Symbol	Gene Name	ID	Fold Change (MGs with ACs/MGs Alone)
Igfbp3	insulin-like growth factor binding protein 3	1423062_at	7877.89
Fscn1	fascin homolog 1, actin bundling protein (Strongylocentrotus purpuratus)	1416514_a_at	1522.45
Igfbp3	insulin-like growth factor binding protein 3	1458268_s_at	948.35
Gja1	gap junction protein, alpha 1	1415800_at	584.05
Ednrb	endothelin receptor type B	1437347_at	559.98
Cnn3	calponin 3, acidic	1436759_x_at	470.72
Wwtr1	WW domain containing transcription regulator 1	1417818_at	379.8
Armcx2	armadillo repeat containing, X-linked 2	1456739_x_at	347.99
Gng11	guanine nucleotide binding protein(G protein), gamma 11	1448942_at	337.17
Lyve1	lymphatic vessel endothelial hyaluronan receptor 1	1429379_at	301.6

## Data Availability

The data underlying this article will be shared upon reasonable request to the corresponding author.

## Supplementary Materials

The following supporting information can be downloaded at: https://www.mdpi.com/article/10.3390/ijms24021774/s1.
