# Supplementary material for: Different Contacted Cell Types Contribute to Acquiring Different Properties in Brain Microglial Cells upon Intercellular Interaction
Source: Int J Mol Sci. 2023 Jan 16;24(2):1774. doi: 10.3390/ijms24021774 (PMC9861207; doi:10.3390/ijms24021774)
Supplement: Supplementary file 1 [file ijms-24-01774-s001.zip › ijms-2129411-supplementary.pdf]

Supplementary Figure S1. Pathway analysis of the cell cycle-related genes between MGs alone and MGs cocultured with ACs.

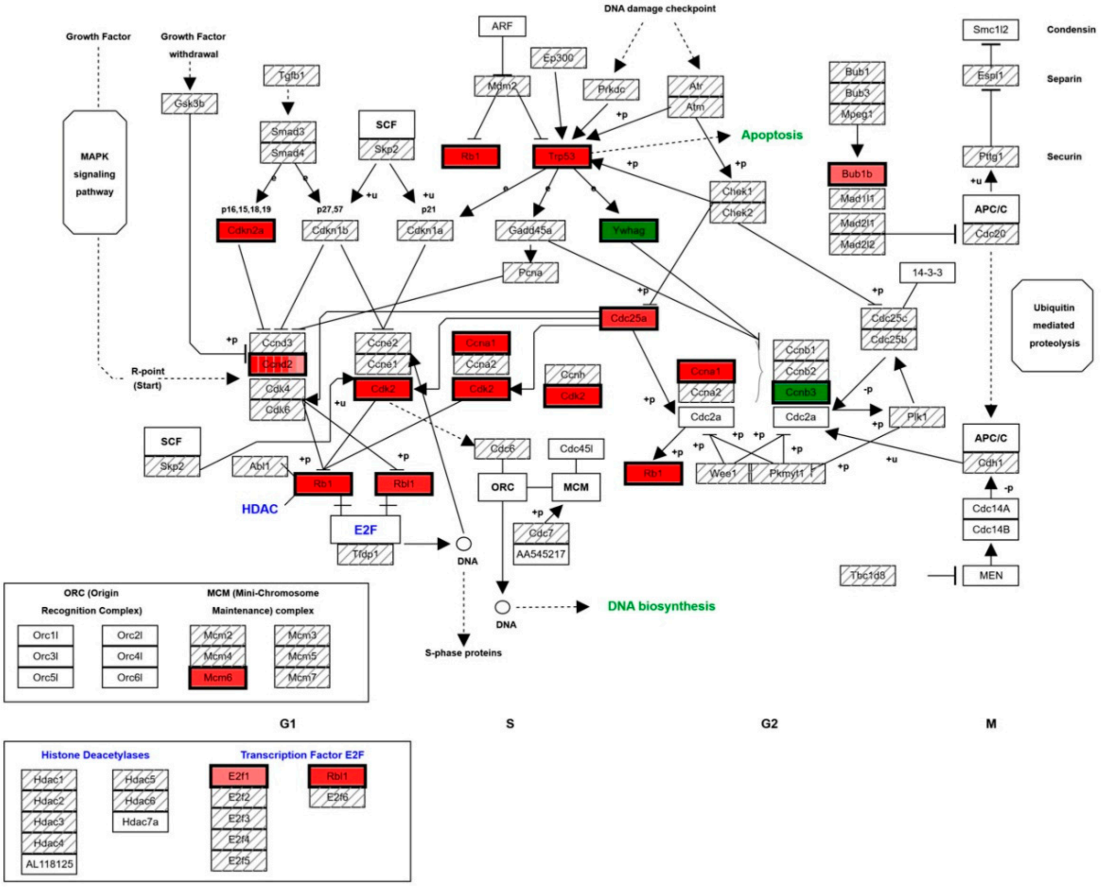

Supplementary Figure S2. Pathway analysis of the cell cycle-related genes between MGs alone and MGs cocultured with PCs.

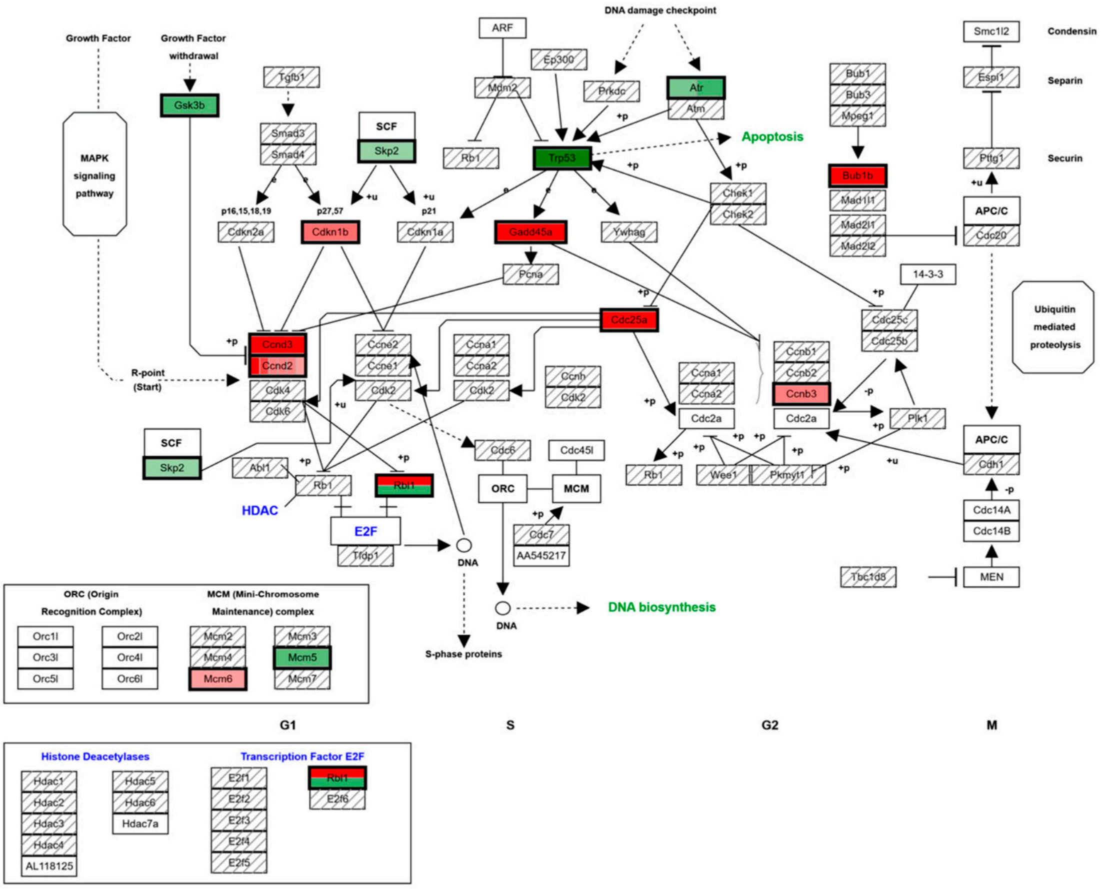

Supplementary Figure S3. Pathway analysis of the cell cycle-related genes between MGs alone and MGs cocultured with ECs.

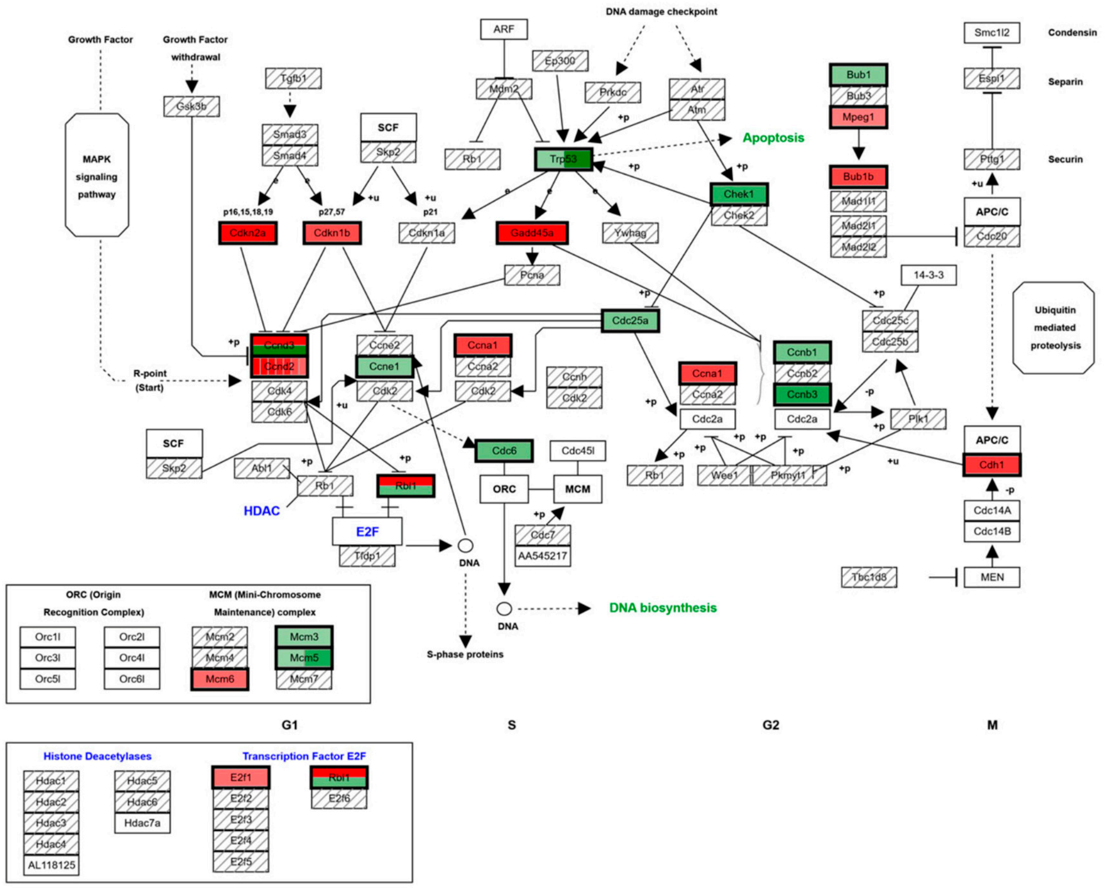

Supplementary Figure S4. Pathway analysis of the apoptosis-related genes between MGs alone and MGs cocultured with ACs.

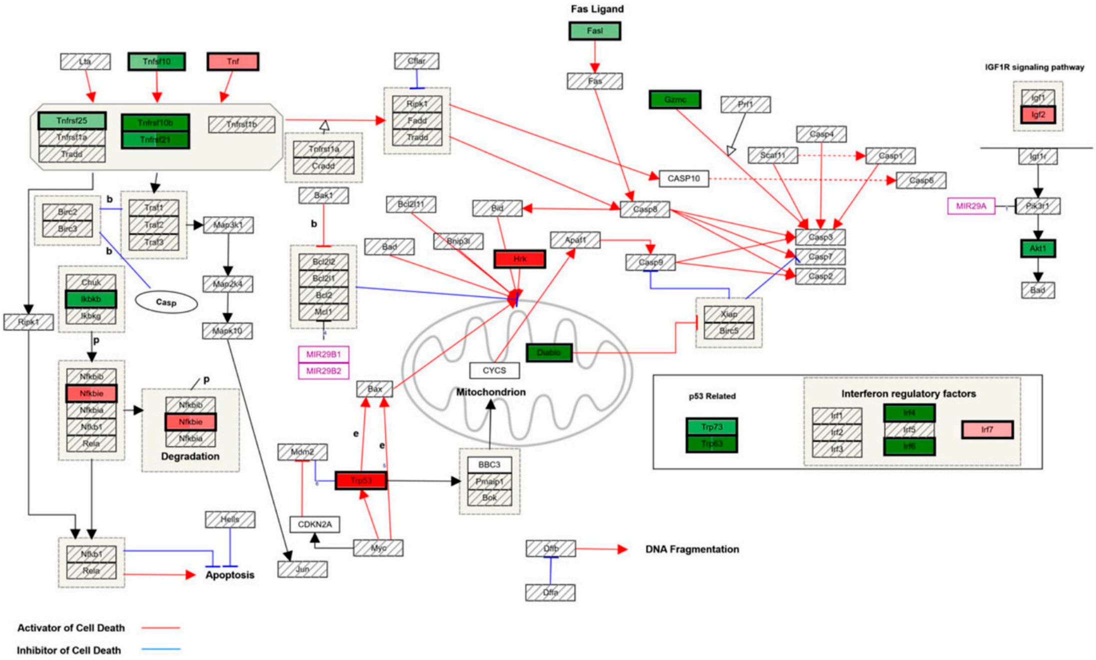

Supplementary Figure S5. Pathway analysis of the apoptosis-related genes between MGs alone and MGs cocultured with PCs.

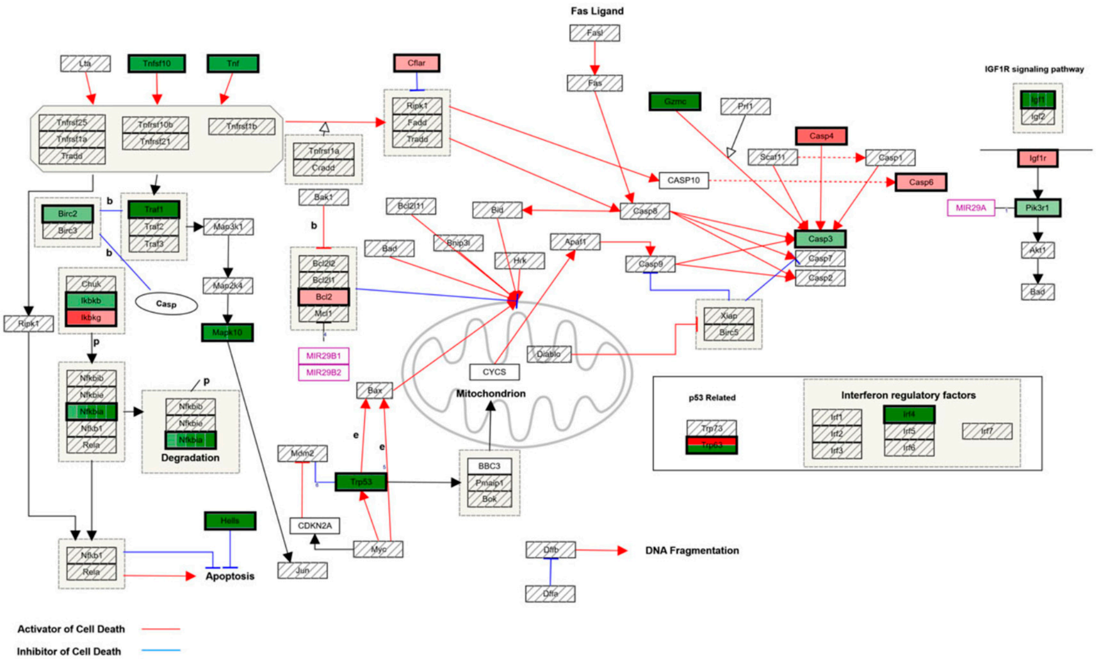

Supplementary Figure S6. Pathway analysis of the apoptosis-related genes between MGs alone and MGs cocultured with ECs.

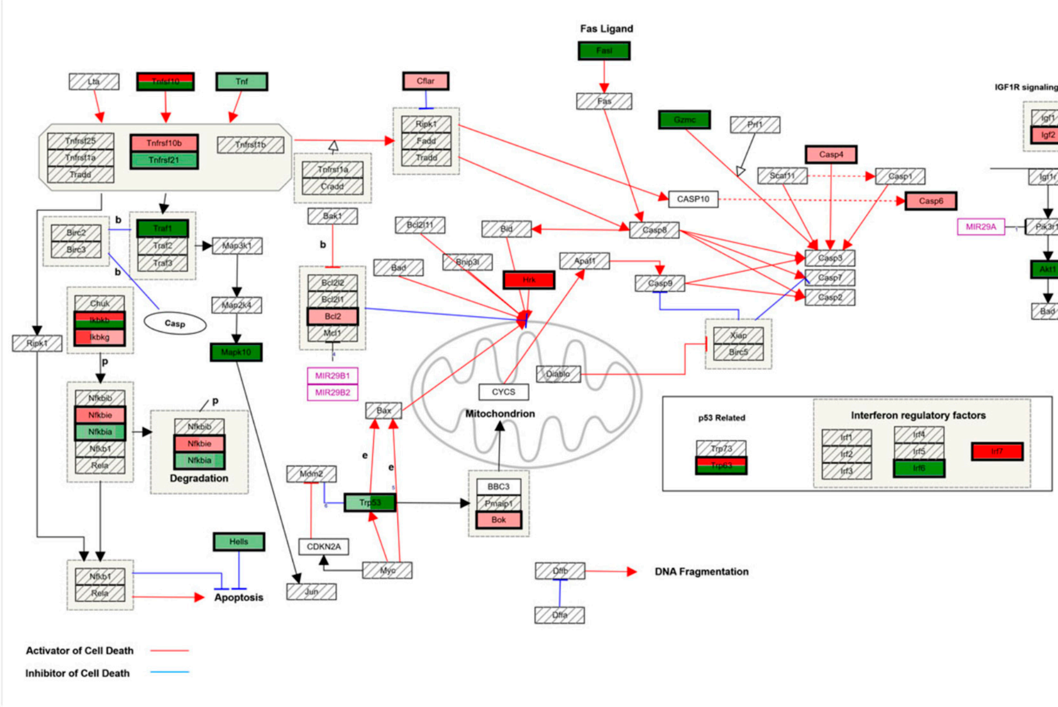

Supplementary Table S1

Comparison of cell cycle-related gene expressions between MGs with ACs and MGs alone (MGs with ACs/MGs alone)

Gene symbol	Gene name	ID	Fold change
Bub1b	budding uninhibited by benzimidazoles 1 homolog, beta (S. cerevisiae)	1447362_at	2.84
Ccna1	cyclin A1	1449177_at	5.45
Ccnb3	cyclin B3	1427744_at	-3.82
Ccnd2	cyclin D2	1430127_a_at	2.29
Cdc25a	cell division cycle 25A	1445995_at	3.46
Cdk2	cyclin-dependent kinase 2	1447617_at	6.94
Cdkn2a	cyclin-dependent kinase inhibitor 2A	1450140_a_at	6.7
E2f1	E2F transcription factor 1	1447840_x_at	2.65
Mcm6	minichromosome maintenance deficient 6 (MIS5 homolog, S. pombe) (S. cerevisiae)	1447756_x_at	3.44
Rb1	retinoblastoma 1	1444400_at	4.19
Rbl1	retinoblastoma-like 1 (p107)	1432605_at	3.59
Trp53	transformation related protein 53	1459780_at	4.17
Ywhag	tyrosine 3-monooxygenase/tryptophan 5-monooxygenase activation protein, gamma polypeptide	1459693_x_at	-17.67

Supplementary Table S2

Comparison of cell cycle-related gene expressions between MGs with PCs and MGs alone (MGs with PCs/MGs alone)

Gene symbol	Gene name	ID	Fold change
Atr	ataxia telangiectasia and Rad3 related	1438921_at	-2.67
Bub1b	budding uninhibited by benzimidazoles 1 homolog, beta (S. cerevisiae)	1447362_at	4.2
Ccnb3	cyclin B3	1427744_at	2.5
Ccnd2	cyclin D2	1416124_at	4.91
Ccnd3	cyclin D3	1444323_at	6.69
Cdc25a	cell division cycle 25A	1445995_at	3.81
Cdkn1b	cyclin-dependent kinase inhibitor 1B	1434045_at	2.69
Gadd45a	growth arrest and DNA-damage-inducible 45 alpha	1449519_at	7.16
Gsk3b	glycogen synthase kinase 3 beta	1439931_at	-2.61
Mcm5	minichromosome maintenance deficient 5, cell division cycle 46 (S. cerevisiae)	1459658_at	-2.53
Mcm6	minichromosome maintenance deficient 6 (MIS5 homolog, S. pombe) (S. cerevisiae)	1447756_x_at	2.14
Rbl1	retinoblastoma-like 1 (p107)	1432604_at	-2.8
Rbl1	retinoblastoma-like 1 (p107)	1454293_at	6.12
Skp2	S-phase kinase-associated protein 2 (p45)	1425072_at	-2.01
Trp53	transformation related protein 53	1459781_x_at	-19.76

Supplementary Table S3

Comparison of cell cycle-related gene expressions between MGs with ECs and MGs alone (MGs with ECs/MGs alone)

Gene symbol	Gene name	ID	Fold change
Bub1	budding uninhibited by benzimidazoles 1 homolog, (S. cerevisiae)	1438571_at	-2.19
Bub1b	budding uninhibited by benzimidazoles 1 homolog, beta (S. cerevisiae)	1447362_at	3.15
Ccna1	cyclin A1	1449177_at	3.21
Ccnb1	cyclin B1	1419944_at	-2.32
Ccnb3	cyclin B3	1427744_at	-3.03
Ccnd2	cyclin D2	1416124_at	7.83
Ccnd3	cyclin D3	1444323_at	8.34
Ccnd3	cyclin D3	1438805_at	-5.44
Ccne1	cyclin E1	1416492_at	-2.16
Cdc6	cell division cycle 6	1417019_a_at	-2.47
Cdc25a	cell division cycle 25A	1445995_at	-2.26
Cdh1	cadherin 1	1448261_at	3.32
Cdkn1b	cyclin-dependent kinase inhibitor 1B	1434045_at	3.08
Cdkn2a	cyclin-dependent kinase inhibitor 2A	1450140_a_at	3.87
Chek1	checkpoint kinase 1	1420031_at	-2.8
E2f1	E2F transcription factor 1	1447840_x_at	2.68
Gadd45a	growth arrest and DNA-damage-inducible 45 alpha	1449519_at	3.86
Mcm3	minichromosome maintenance deficient 3 (S. cerevisiae)	1426652_at	-2.05
Mcm5	minichromosome maintenance deficient 5, cell division cycle 46 (S. cerevisiae)	1415945_at	-2.03
Mcm6	minichromosome maintenance deficient 6 (MIS5 homolog, S. pombe) (S. cerevisiae)	1447756_x_at	2.74
Mpeg1	macrophage expressed gene 1	1427076_at	2.54
Rbl1	retinoblastoma-like 1 (p107)	1454293_at	7.38
Rbl1	retinoblastoma-like 1 (p107)	1432604_at	-2.5
Trp53	transformation related protein 53	1438808_at	-2.1

Supplementary Table S4

Comparison of apoptosis-related gene expressions between MGs with ACs and MGs alone (MGs with ACs/MGs alone)

Gene symbol.	Gene name	ID	Fold change
Akt1	thymoma viral proto-oncogene 1	1442759_at	-2.97
Diablo	diablo homolog (Drosophila)	1425768_at	-8.78
Fasl	Fas ligand (TNF superfamily, member 6)	1449235_at	-2.34
Gzmc	granzyme C	1421256_at	-3.54
Hrk	harakiri, BCL2 interacting protein (contains only BH3 domain)	1439854_at	3.64
Igf2	insulin-like growth factor 2	1448152_at	2.49
Ikbkb	inhibitor of kappaB kinase beta	1432275_at	-3.11
Irf4	interferon regulatory factor 4	1421174_at	-4.35
Irf6	interferon regulatory factor 6	14180301_at	-26.88
Irf7	interferon regulatory factor 7	1417244_a_at	2
Nfkbie	nuclear factor of kappa light polypeptide gene enhance in B cells inhibitor, epsilon	1458299_s_at	2.93
Tnf	tumor necrosis factor	1419607_at	2.48
Tnfrsf10b	tumor necrosis factor receptor superfamily, member 10b	1421296_at	-3.61
Tnfrsf21	tumor necrosis factor receptor superfamily, member 21	1450731_s_at	-9.03
Tnfrsf25	tumor necrosis factor receptor superfamily, membrane 25	1422231_a_at	-2.26
Tnfsf10	tumor necrosis factor (ligand) superfamily, member 10	1439680_at	-3.14
Trp53	transformation related protein 53	1459780_at	4.17
Trp63	transformation related protein 63	1452027_a_at	-5.77
Trp73	transformation related protein 73	1452325_at	-2.89

Supplementary Table S5

Comparison of apoptosis-related gene expressions between MGs with PCs and MGs alone (MGs with PCs/MGs alone)

Gene symbol	Gene name	ID	Fold change
Bcl2	B cell leukemia/lymphoma 2	1457687_at	2.03
Birc2	baculoviral IAP repeat-containing 2	1430752_at	-2.37
Casp3	caspase 3	1426165_a_at	-2.3
Casp4	caspase 4, apoptosis-related cysteine peptidase	1449591_at	2.79
Casp6	caspase 6	1458715_at	2.12
Cflar	CASP8 and FADD-like apoptosis regulator	1424996_at	2.07
Gzmc	granzyme C	1421256_at	-10.32
Hells	helicase, lymphoid specific	1453361_at	-3.88
Igf1	insulin-like growth factor 1	1419519_at	-5.03
Igf1r	insulin-like growth factor 1 receptor	1452108_at	2.27
Ikbkb	inhibitor of kappaB kinase beta	1432275_at	-2.67
Ikbkg	inhibitor of kappaB kinase gamma	1421208_at	2.19
Irf4	interferon regulatory factor 4	1421174_at	-5.69
Mapk10	mitogen-activated protein kinase 10	1448342_at	-3.41
Nfkbia	nuclear factor of kappa light polypeptide gene enhancer in B cells inhibitor, alpha	1420089_at	-2.68
Pik3r1	phosphatidylinositol 3-kinase, regulatory subunit, polypeptide 1 (p85 alpha)	1425514_at	-2.05
Tnf	tumor necrosis factor	1419607_at	-3.15
Tnfsf10	tumor necrosis factor (ligand) superfamily, member 10	1420412_at	-3.15
Traf1	TNF receptor-associated factor 1	1445452_at	-11.02
Trp53	transformation related protein 53	1459781_x_at	-19.76
Trp63	transformation related protein 63	1459581_at	8.67
Trp63	transformation related protein 63	1451876_a_at	-4.15

Supplementary Table S6

Comparison of apoptosis-related gene expressions between MGs with ECs and MGs alone (MGs with ECs/MGs alone)

Gene symbol	Gene name	ID	Fold change
Akt1	thymoma viral proto-oncogene 1	1442759_at	-6.83
Bcl2	B cell leukemia/lymphoma 2	1437122_at	2.04
Bok	BCL2-related ovarian killer	1417040_a_at	2.15
Casp4	caspase 4, apoptosis-related cysteine peptidase	1449591_at	2.14
Casp6	caspase 6	1458715_at	2.29
Cflar	CASP8 and FADD-like apoptosis regulator	1424996_at	2.02
Fasl	Fas ligand (TNF superfamily, member 6)	1418803_a_at	-4.49
Gzmc	granzyme C	1421256_at	-5.29
Hells	helicase, lymphoid specific	1453361_at	-2.35
Hrk	harakiri, BCL2 interacting protein (contains only BH3 domain)	1439854_at	4.3
Igf2	insulin-like growth factor 2	1448152_at	2.42
Ikbkb	inhibitor of kappaB kinase beta	1445141_at	3.39
Ikbkb	inhibitor of kappaB kinase beta	1432275_at	-3.96
Ikbkg	inhibitor of kappaB kinase gamma	1421208_at	2.12
Irf6	interferon regulatory factor 6	1418301_at	-3.56
Irf7	interferon regulatory factor 7	1417244_a_at	5.3
Mapk10	mitogen-activated protein kinase 10	1448342_at	-11.27
Nfkbia	nuclear factor of kappa light polypeptide gene enhancer in B cells inhibitor, alpha	1420089_at	-2.29
Nfkbie	nuclear factor of kappa light polypeptide gene enhancer in B cells inhibitor, epsilon	1458299_s_at	2.61
Tnf	tumor necrosis factor	1419607_at	-2.28
Tnfsf10	tumor necrosis factor (ligand) superfamily, member 10	1420412_at	4.6
Tnfsf10	tumor necrosis factor (ligand) superfamily, member 10	1439680_at	-4.22
Tnfrsf10b	tumor necrosis factor receptor superfamily, member 10b	1422344_s_at	2.48
Tnfrsf21	tumor necrosis factor receptor superfamily, member 21	1450731_s_at	-2.66
Traf1	TNF receptor-associated factor 1	1445452_at	-6.57
Trp53	transformation related protein 53	1459781_x_at	-20.55
Trp63	transformation related protein 63	1459581_at	3.79
Trp63	transformation related protein 63	1452027_a_at	-5.0

Supplementary Table S7

Comparison of MG-related gene expressions between MGs with ACs and MGs alone
(MGs with ACs/MGs alone)

Gene symbol	Gene name	ID	Fold change
Cd68	CD68 antigene	1449164_at	-1.02
Cd80	CD80 antigen	1451950_a_at	1.55
Cd86	CD86 antigen	1420404_at	1.61
Cd163	CD163 antigen	1419144_at	1.86
Csf1r	colony stimulating factor 1 receptor	1419872_at	1.08
Itgam	integrin alpha M	1422046_at	1.1
Mrc1	mannose receptor, C type 1	1450430_at	1.42
Trem2	triggering receptor expressed on myeloid cells 2	1421792_s_at	1.06
Tmem119	transmembrane protein 119	1451344_at	1.72
P2ry12	purinergic receptor P2Y, G-protein coupled 12	1431724_a_at	3.83

Supplementary Table S8

Comparison of MG-related gene expressions between MGs with PCs and MGs alone (MGs with PCs/MGs alone)

Gene symbol	Gene name	ID	Fold change
Cd68	CD68 antigene	1449164_at	-1.07
Cd80	CD80 antigen	1451950_a_at	1.37
Cd86	CD86 antigen	1420404_at	1.74
Cd163	CD163 antigen	1419144_at	-2.5
Csf1r	colony stimulating factor 1 receptor	1419872_at	-1.37
Itgam	integrin alpha M	1422046_at	1.4
Mrc1	mannose receptor, C type 1	1450430_at	1.08
Trem2	triggering receptor expressed on myeloid cells 2	1421792_s_at	-1.05
Tmem119	transmembrane protein 119	1451344_at	2.76
P2ry12	purinergic receptor P2Y, G-protein coupled 12	1431724_a_at	1.28

Supplementary Table S9

Comparison of MG-related gene expressions between MGs with ECs and MGs alone (MGs with ECs/MGs alone)

Gene symbol	Gene name	ID	Fold change
Cd68	CD68 antigene	1449164_at	1.03
Cd80	CD80 antigen	1451950_a_at	1.14
Cd86	CD86 antigen	1420404_at	1.69
Cd163	CD163 antigen	1419144_at	3.77
Csf1r	colony stimulating factor 1 receptor	1419872_at	1.17
Itgam	integrin alpha M	1422046_at	1.35
Mrc1	mannose receptor, C type 1	1450430_at	3.09
Trem2	triggering receptor expressed on myeloid cells 2	1421792_s_at	1.1
Tmem119	transmembrane protein 119	1451344_at	1.86
P2ry12	purinergic receptor P2Y, G-protein coupled 12	1431724_a_at	2.24
